# Total cholesterol variability and risk of atrial fibrillation: A nationwide population-based cohort study

**DOI:** 10.1371/journal.pone.0215687

**Published:** 2019-04-24

**Authors:** Eun Roh, Hye Soo Chung, Ji Sung Lee, Jung A. Kim, You-Bin Lee, So-hyeon Hong, Nam Hoon Kim, Hye Jin Yoo, Ji A. Seo, Sin Gon Kim, Nan Hee Kim, Sei Hyun Baik, Kyung Mook Choi

**Affiliations:** 1 Division of Endocrinology and Metabolism, Department of Internal Medicine, Korea University College of Medicine, Seoul, Korea; 2 Division of Endocrinology and Metabolism, Department of Internal Medicine, Hallym University College of Medicine, Seoul, Korea; 3 Clinical Research Center, Asan Medical Center, University of Ulsan College of Medicine, Seoul, Korea; Medizinische Universitat Innsbruck, AUSTRIA

## Abstract

**Background:**

Long-term variability of cardiometabolic risk factors have been suggested as the risk factors for cardiovascular disease and mortality. However, the effect of long-term variability of total cholesterol (TC) on incident atrial fibrillation (AF) has not been examined.

**Methods and findings:**

We explored whether visit-to-visit TC variability are associated with the risk of incident AF in 160,165 Korean adults, using the population-based Korean National Health Insurance Service–Health Screening Cohort (NHIS-HEALS) database, over a median duration of 8.4 years. TC variability was measured as coefficients of variance (TC-CV), standard deviation (TC-SD), and variability independent of the mean (TC-VIM). Kaplan–Meier analysis demonstrated a decreased disease-free probability in the highest quartile group of TC variability compared to that in the other groups. In the multivariate Cox proportional hazard analysis, the risk of AF increased significantly in the highest quartile group of TC variability. After multivariate adjustment for confounding variables including mean TC levels, the hazard ratio for incident AF was 1.15 (95% confidence interval 1.05–1.25; *P* = 0.0035) when comparing the highest with the lowest TC variability quartile (TC-CV). These relationships were consistent with TC variability defined using TC-SD or TC-VIM. Subgroup analyses, including age, sex, body mass index, and cardiometabolic disorders, showed similar results.

**Conclusions:**

The present study is the first to demonstrate that high TC variability was associated with an increased risk of AF.

## 1. Introduction

A worldwide epidemic of atrial fibrillation (AF) is nowadays recognized as a pivotal health threat. Previous studies have shown that AF is associated with an increased risk of stroke, cardiovascular disease (CVD), and mortality [1]. Park et al. reported that the prevalence of AF is 1.3% and that it increases with age in Korea [2]. In a Chinese population aged over 60 years, the prevalence of AF was 2.0% in men and 1.6% in women [3], and the total number of individuals with AF in China had exceeded 5 million in 2010 due to the large population size [4]. In the United States, the prevalence of AF was 1.1% in men and 0.8% in women, and it is estimated that the number of patients with AF will increase 2.5-fold to more than 5.6 million by 2050 [5]. In the age stratum of 55–59 years, the prevalence of AF was 1.3% in men and 1.7% in women, and the number of patients aged ≥55 years with AF will double by 2060 in the European Union [6].

Previous studies have identified several risk factors for the development of AF. The dominant role of increasing age on the development of AF is well recognized [7, 8]. Men usually present with higher incidence of AF than women across all age groups [8]. Hypertension is the most important risk factor for AF due to its high prevalence [1]. Heart failure and valvular heart disease, which lead to atrial pressure and/or volume overload and ventricular dysfunction, are associated with AF [8]. Obesity, which is linked with metabolically active epicardial fat tissue, has been identified as a risk factor for AF based on epidemiological and clinical evidence [9]. The Framingham cohort study found that risk factors for CVD, such as diabetes and hypertension, are predisposing factors for AF [10].

Recently, several studies have demonstrated that long-term variability of cardiometabolic risk factors, such as blood pressure, glucose, and cholesterol, is associated with increased risk of CVD and mortality [11–13]. Kim et al. reported that high total cholesterol (TC) variability is associated with all-cause mortality, myocardial infarction, and stroke in the Korean population [14]. Although the overall prevalence of hypercholesterolemia increased over the past decade, treatment rate was only 7.4% in 2010 and only 30% of dyslipidemic patients who received lipid-lowering treatment reached target levels in Korea [15]. The low control rate of hypercholesterolemia may increase the long-term TC variability [16]. Therefore, we examined the effect of visit-to-visit variability of TC on the incidence of AF using the longitudinal National Health Insurance Service-National Health Screening Cohort (NHIS-HEALS) database.

## 2. Materials and methods

### 2.1 Data source and study population

The NHIS is a mandatory social health insurance program that covers almost the entire (approximately 98%) Korean population who participate biannually in standardized health examinations provided by the Korean government [17, 18]. The database established by NIHS includes an eligibility database (included data such as age, sex, and socioeconomic variables), a health examination database (comprising questionnaires on the health-related behavioral variables and the results of laboratory measurements), and a medical history database (comprising information on diagnosis, medication, admission, and death). Anthropometric and laboratory measurements were performed after an overnight fast. Quality control procedures were checked by the Korean Association of Laboratory Quality Control. The NHIS-HEALS database was used to randomly select approximately 10% of the entire participants within the NHIS database who were aged from 40 to 79 years.

**Fig 1:**
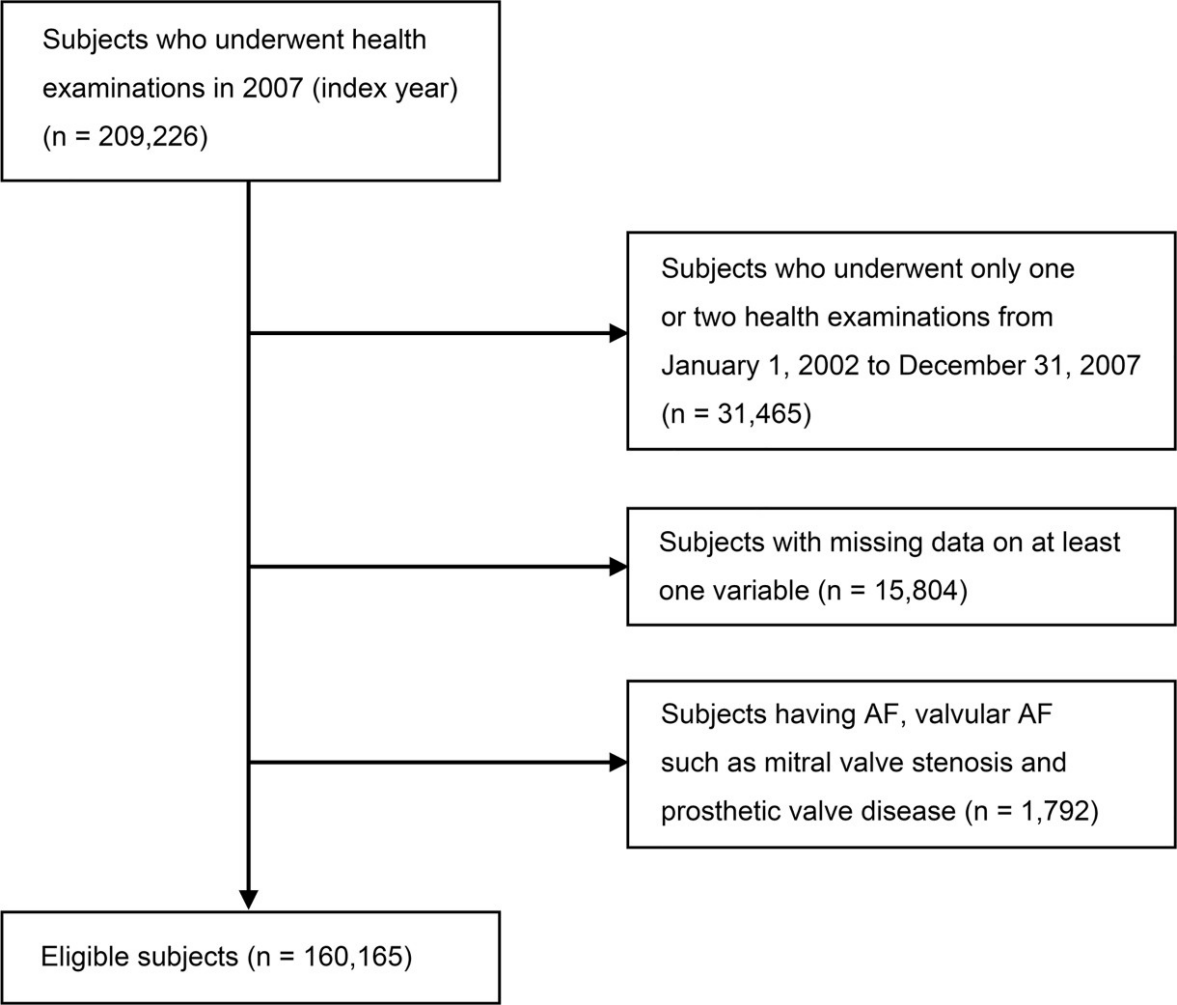
Flowchart of the study population.

In this study, we used the data of participants who underwent the national health examinations in 2007 (index year), and three or more health examinations from January 1, 2002 to December 31, 2007. A total of 31,465 participants who underwent only one or two examinations and 15,804 participants with missing data on at least one variable were excluded. An additional 1,792 patients were excluded due to a previous diagnosis of AF, valvular AF, such as mitral valve stenosis and prosthetic valve disease based on diagnoses coded according to the International Classification of Diseases, 10th revision (ICD‐10) and questionnaires about medical history before the index year. A total 160,165 individuals were finally included in the analysis (Fig 1). The protocols were approved by the NHIS review committee, and the Korea University institutional review board approved the study protocol in accordance with the Declaration of Helsinki of the World Medical Association (IRB No. KUGH16118-001). Informed consent was waived because anonymous and de-identified information was used for analysis.

### 2.2 Assessment and definitions

Body mass index (BMI) was calculated as weight in kilograms (kg) divided by the square of height in meters (m^2^). Smoking status and information concerning alcohol consumption were obtained from a questionnaire filled-in during the health examination. Regular exercise was defined as strenuous physical activity for at least 20 min and ≥5 times/week. Income levels were dichotomized at the lower 10%. The presence of diabetes was defined based on the criteria of fasting glucose levels ≥126 mg/dL or the presence of at least one claim per year for the prescription of antidiabetic medication under the ICD-10 codes (E10–E14). The presence of hypertension was defined based on the criteria of systolic/diastolic blood pressure ≥140/90 mmHg or the presence of at least one claim per year for the prescription of an antihypertensive agent under ICD-10 codes (I10–I15). The presence of dyslipidemia was defined based on the criteria of TC levels ≥240 mg/dL or the presence of at least one claim per year for the prescription of an antihyperlipidemic agent under ICD-10 codes (E78). The presence of heart failure was defined using ICD-10 codes (I50) during hospitalization. Other co-morbidities were assessed by searching for ICD-10 codes: ischemic heart disease (I20-25), cerebrovascular disease (I60-69, G45), heart valvular disease (I05, I34-37, Z95), chronic kidney disease (CKD) (N18), thyroid disorders (E03, E05, E89), chronic obstructive pulmonary disease (COPD) (J43-44), and obstructive sleep apnea (OSA) (G47.3).

Visit-to-visit TC variability was determined from at least three measurements of TC values for each participant: 3 measurements for 80,894 participants (50.5%), 4 measurements for 17,348 participants (10.8%), 5 measurements for 23,178 participants (14.5%), and 6 measurements for 38,745 participants (24.2%). For a description of TC variability, we used three indices of variability, namely the coefficient of variation (TC-CV), standard deviation (TC-SD), and variability independent of the mean (TC-VIM). The CV was defined as the SD divided by the mean (SD/mean) × 100 (%) and the VIM was defined as 100 × SD/Mean^β^, where β is the regression coefficient, based on the natural logarithm of the SD divided by the natural logarithm of the mean.

AF was diagnosed using the ICD-10 code I48. We examined newly occurring AF cases in the governmental database from January 1, 2008 to December 31, 2015. Patients were defined as having AF when it was a discharge diagnosis or confirmed at least twice in the outpatient department. The AF diagnosis has been validated previously with a positive predictive value of 94.1% [19].

### 2.3 Statistical analysis

Data were analyzed using SAS 9.4 (SAS Institute Inc., Cary, NC, USA). Statistical significance was assumed at *P* <0.05. All statistical analyses were performed by an experienced professional statistician, who was also one of authors. Results are presented as the mean ± SD for continuous variables and counts or percentages (%) for categorical variables. Participants were classified into four groups according to quartiles of the TC variability. Statistical analysis for between-group comparisons was based on analysis of variance (ANOVA) for continuous variables or the χ^2^ test for categorical variables.

Kaplan–Meier curves for disease-free probability of AF were obtained for the four groups, which were classified and expressed as quartiles of TC variability. Hazard ratios (HRs) and 95% confidence interval (CI) values for the risk of AF were analyzed using Cox proportional hazards models for quartile groups of TC variability after adjusting for the multiple confounding factors. We tested the assumption of proportionality of hazards using the numerical method proposed by Lin et al., derived from cumulative sums of martingale-based residuals [20]. We found no evidence of violating the proportional hazards assumption.

Subgroup analysis was conducted by stratification for age, sex, BMI, smoking, alcohol intake, exercise, income, hypertension, dyslipidemia, diabetes, heart failure, ischemic heart disease, cerebrovascular disease, CKD, thyroid disorders, COPD, and OSA and the use of antihypertensive or antidyslipidemic agents. In subgroup analyses, the HR and 95% CI of the highest quartile (Q4) group were compared with those of the lower three quartiles (Q1-Q3) as the reference group using Cox proportional hazards regression models with interaction effect. Because of post hoc subgroup analyses, we did not adjust for multiple testing.

## 3. Results

### 3.1 Baseline characteristics

Among the 160,165 subjects with complete follow-up data, 3,884 (2.4%) had incident AF events during the median follow-up period of 8.4 years. The baseline characteristics of the study subjects according to the presence of AF was shown in S1 Table. Subjects with AF were older and predominantly male. They had higher BMIs, elevated blood pressure, and elevated fasting plasma glucose levels. Subjects with AF had more comorbidities, such as hypertension, diabetes, dyslipidemia, heart failure, ischemic heart disease, cerebrovascular disease, CKD, thyroid disorders, COPD, and OSA. Mean TC levels were significantly lower in subjects with AF (198.9±30.4 vs. 196.4±30.7, *P* <0.0001). TC variability measured as CV, SD, and VIM was significantly higher in subjects with AF.

Table 1 shows the baseline characteristics of the study population according to the quartiles of TC variability (TC-CV). Higher quartile groups of TC variability showed elevated blood pressure and elevated levels of fasting plasma glucose. They had lower incomes and higher prevalence of hypertension, dyslipidemia, diabetes, heart failure, ischemic heart disease, cerebrovascular disease, and thyroid disorders. The baseline characteristics of study subjects were similar when TC variability was expressed by SD and VIM (S2 Table and S3 Table).

**Table 1:** Baseline characteristics of subjects according to the total cholesterol variability.

	Q1	Q2	Q3	Q4	P-value
N	40041	40041	40042	40041	
Age (years)	55.9±8.8	54.9±8.3	55.4±8.5	57.4±9.1	< .0001
Sex (male) (n, %)	23251 (58.1)	25107 (62.7)	24164 (60.3)	21790 (54.4)	< .0001
Body mass index (kg/m2)	23.9±2.9	23.9±2.8	23.9±2.8	24.1±2.9	< .0001
Systolic BP (mmHg)	125.2±15.6	125.4±15.5	125.7±15.7	126.6±16.1	< .0001
Diastolic BP (mmHg)	78.0±10.2	78.4±10.2	78.5±10.2	78.5±10.3	< .0001
Aspartate transaminase (IU/L)	25.5±12.3	25.7±13.9	26.2±15.6	27.6±20.4	< .0001
Alanine transaminase (IU/L)	24.4±16.4	24.9±19.3	25.3±19.0	26.5±22.3	< .0001
γ-glutamyl transferase (IU/L)	35.2±41.1	37.4±44.2	38.8±49.5	42.7±63.5	< .0001
Fasting plasma glucose (mmol/L)	97.9±23.6	97.9±23.9	98.8±25.9	101.3±29.5	< .0001
Mean TC (mg/dL)	199.3±30.0	198.6±29.8	197.7±30.1	199.6±31.9	< .0001
TC variability					
TC-CV (%)	4.36±1.43	7.77±0.82	10.94±1.08	18.17±5.49	< .0001
TC-SD (IU/L)	8.68±3.15	15.44±2.85	21.63±3.94	36.36±13.72	< .0001
TC-VIM (%)	8.65±2.89	15.41±1.91	21.64±2.56	36.11±11.59	< .0001
Current smoker (n, %)	7788 (19.5)	8728 (21.8)	8488 (21.2)	7534 (18.8)	< .0001
Alcohol consumption (n, %)	17194 (42.9)	18288 (45.7)	17646 (44.1)	15656 (39.1)	< .0001
Regular exercise (n, %)	4252 (10.6)	3924 (9.8)	3949 (9.9)	4325 (10.8)	< .0001
Income (lower 10%) (n, %)	2934 (7.3)	2995 (7.5)	3278 (8.2)	3593 (9.0)	< .0001
Hypertension (n, %)	22560 (56.3)	23345 (58.3)	23985 (59.9)	26165 (65.3)	< .0001
Dyslipidemia (n, %)	8717 (21.8)	11140 (27.8)	13895 (34.7)	22017 (55.0)	< .0001
Diabetes (n, %)	5772 (14.4)	6278 (15.7)	7068 (17.7)	9318 (23.3)	< .0001
Heart failure (n, %)	804 (2.0)	775 (1.9)	835 (2.1)	1422 (3.6)	< .0001
Ischemic heart disease (n, %)	4807 (12.0)	4583 (11.4)	4934 (12.3)	7497 (18.7)	< .0001
Cerebrovascular disease (n, %)	3418 (8.5)	3191 (8.0)	3649 (9.1)	5221 (13.0)	< .0001
Chronic kidney disease (n, %)	156 (0.4)	151 (0.4)	150 (0.4)	315 (0.8)	< .0001
Thyroid disorder (n, %)	2750 (6.9)	2662 (6.6)	3018 (7.5)	4107 (10.3)	< .0001
Chronic obstructive pulmonary disease (n, %)	2446 (6.1)	2087 (5.2)	2424 (6.1)	3137 (7.8)	< .0001
Obstructive sleep apnea (n, %)	106 (0.3)	135 (0.3)	136 (0.3)	148 (0.4)	0.0630

*P* value derived using ANOVA and χ^2^ tests. Data are expressed as mean ± SD, or n (%). Abbreviations: BP, blood pressure; CV, coefficients of variance; SD, standard deviation; TC, total cholesterol; VIM, variability independent of the mean.

### 3.2 Impact of TC variability on AF

**Fig 2:**
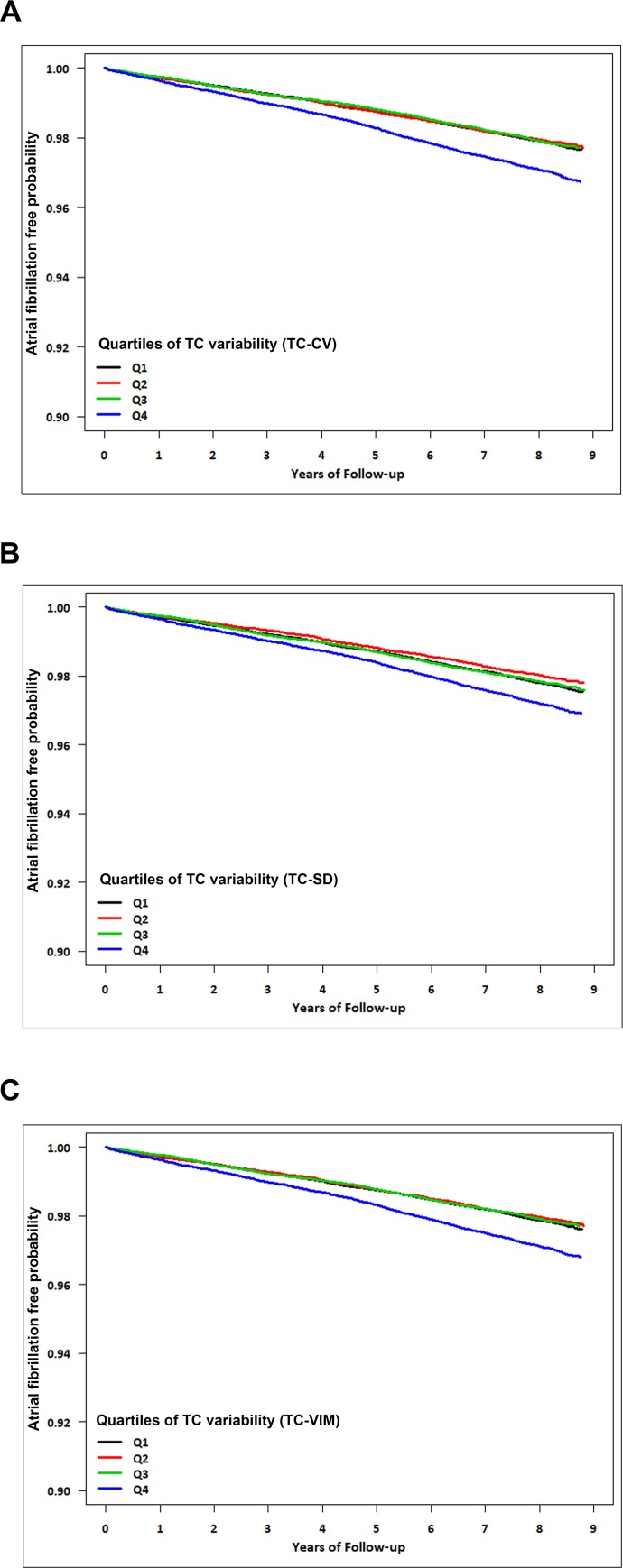
Kaplan–Meier estimates of disease-free probability of AF by quartiles of TC variability. (A) TC-CV, (B) TC-SD, (C) TC-VIM Abbreviations: AF, atrial fibrillation; CV, coefficients of variance; SD, standard deviation; TC, total cholesterol; VIM, variability independent of the mean.

Fig 2 illustrates the Kaplan-Meier curves assessing AF in patients stratified across quartiles of TC variability. Kaplan–Meier analysis demonstrated a decreased disease-free probability in the highest quartile group of TC variability compared to that in the other groups. There were no significant differences to AF risk between subjects in the lower three quartiles. In the multivariate Cox proportional hazard analysis, the risk of AF increased significantly in the highest quartile group of TC variability (Q4) compared with those within lower three quartiles (Q1-Q3) (Table 2). HR for the highest versus lowest quartile (Q4 vs. Q1) of TC variability was 1.15 (95% CI 1.05–1.25, *P* = 0.0035 for TC-CV), 1.11 (95% CI 1.01–1.22, *P* = 0.0235 for TC-SD), and 1.14 (95% CI 1.04–1.25, *P* = 0.0044 for TC-VIM).

**Table 2:** Hazard ratios and 95% confidence intervals of atrial fibrillation by quartiles of total cholesterol variability.

	Events (n)	Follow-up duration (person years)	Incidence rate (per 1000 person years)	†Adjusted HR (95% CI)	*P* value
TC variability (CV)					
Q1	893	333703	2.68	1 (ref)	
Q2	863	333423	2.59	1.03 (0.94–1.13)	0.4972
Q3	885	333459	2.65	1.00 (0.91–1.10)	0.9257
Q4	1243	332745	3.74	1.15 (1.05–1.25)	0.0035
*P* for trend				0.0076	
TC variability (SD)					
Q1	936	333524	2.81	1 (ref)	
Q2	843	333606	2.53	0.97 (0.88–1.06)	0.4726
Q3	915	333167	2.75	1.01 (0.92–1.11)	0.7882
Q4	1190	333033	3.57	1.11 (1.01–1.22)	0.0235
*P* for trend				0.0158	
TC variability (VIM)					
Q1	907	333662	2.72	1 (ref)	
Q2	862	333456	2.59	1.02 (0.93–1.12)	0.7318
Q3	886	333415	2.66	1.00 (0.91–1.09)	0.9426
Q4	1229	332798	3.69	1.14 (1.04–1.25)	0.0044
*P* for trend				0.0084	

†Adjusted for age, sex, income, BMI, hypertension, dyslipidemia, diabetes, smoking, alcohol intake, exercise, heart failure, ischemic heart disease, cerebrovascular disease, chronic kidney disease, thyroid disorder, chronic obstructive pulmonary disease, obstructive sleep apnea, and mean total cholesterol.

Abbreviations: CI, confidence interval; CV, coefficients of variance; HR, hazard ratio; SD, standard deviation; TC, total cholesterol; VIM, variability independent of the mean.

**Fig 3:**
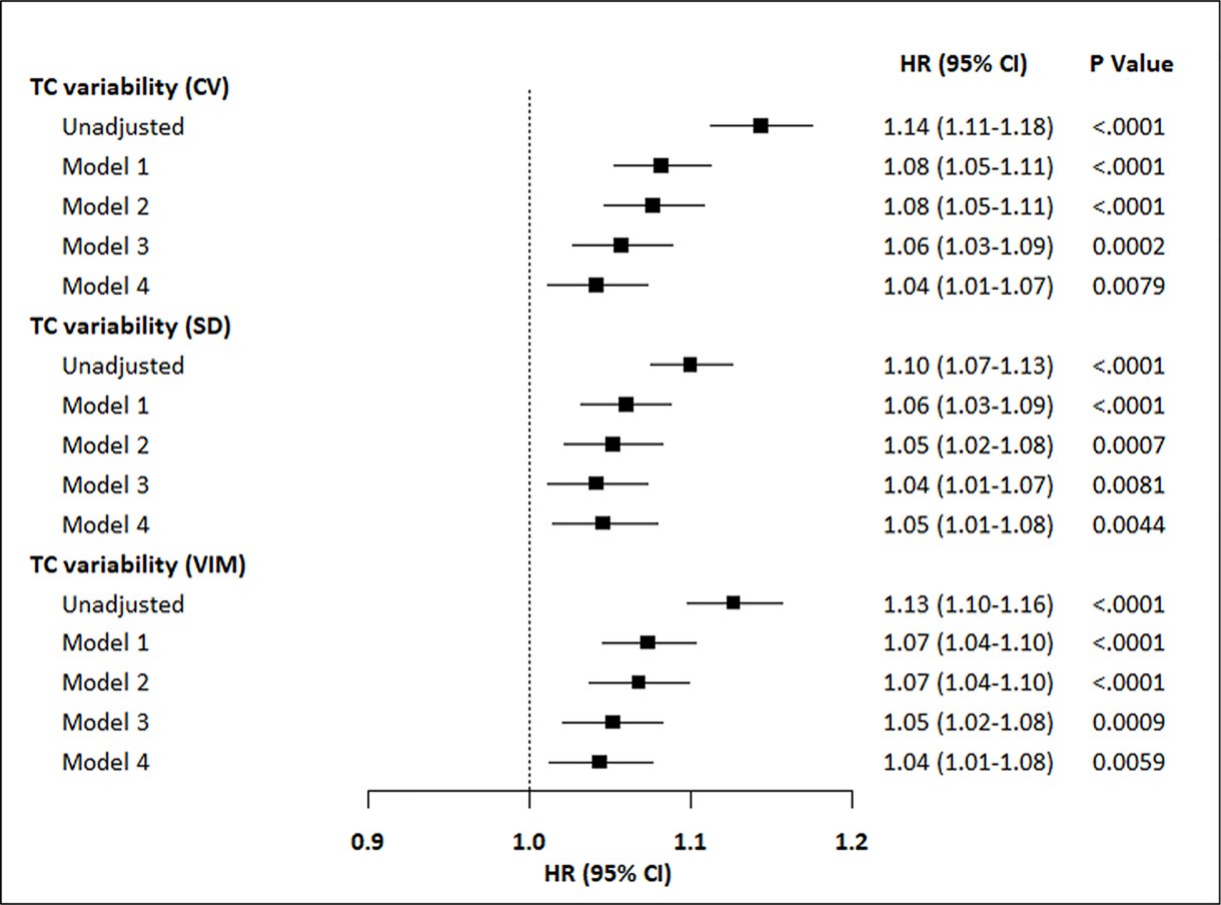
Increase in the risk of AF for every 1-SD increase in TC variability in the unadjusted model and models adjusted for risk factors including mean TC level. Model 1: Adjusted for age, sex Model 2: model 1 + income, BMI, hypertension, dyslipidemia, diabetes, smoking, alcohol intake, exercise Model 3: model 2 + heart failure, ischemic heart disease, cerebrovascular disease, chronic kidney disease, thyroid disorder, chronic obstructive pulmonary disease, obstructive sleep apnea Model 4: model 3 + mean total cholesterol Abbreviations: AF, atrial fibrillation; CI, confidence interval; CV, coefficients of variance; HR, hazard ratios; SD, standard deviation; TC, total cholesterol; VIM, variability independent of the mean.

**Fig 4:**
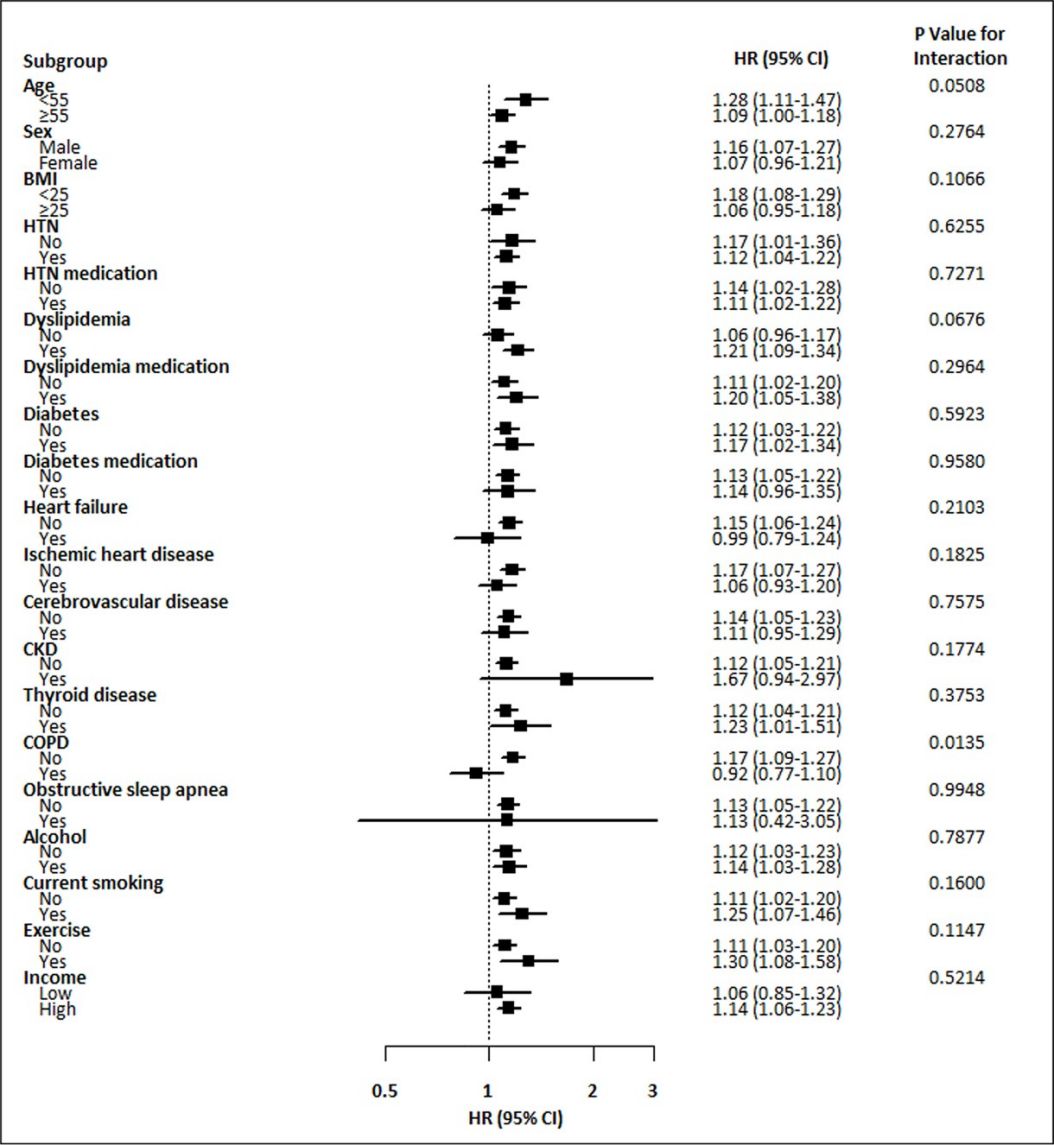
Hazard ratios and 95% confidence intervals for the risk of AF in the highest quartile vs. lower three quartiles of TC variability (TC-CV) in subgroups. Abbreviations: AF, atrial fibrillation; BMI, body mass index; CKD, chronic kidney disease; COPD, chronic obstructive pulmonary disease; CV, coefficients of variance; HTN, hypertension; TC, total cholesterol.

For every 1-SD increase in TC variability, the risk of AF events increased by 14% (95% CI 11–18%, *P* <0.0001 for TC-CV), 10% (95% CI 7–13%, *P* <0.0001 for TC-SM), and 13% (95% CI 10–16%, *P* <0.0001 for TC-VIM) (Fig 3). Adjustment for traditional risk factors and comorbidities did not attenuate the positive association of the increase in TC variability with AF. Moreover, the association remained significant even after adjustment for mean TC. Stratified analyses were conducted by age, sex, BMI, smoking, alcohol intake, income and presence or absence of diabetes, hypertension, dyslipidemia, heart failure, ischemic heart disease, cerebrovascular disease, CKD, thyroid disorders, COPD, and OSA and the use of antihypertensive or antidyslipidemic agents (Fig 4). TC-CV Q4 remained predictive of AF in almost all subgroups when compared with Q1-Q3 without significant interaction. Higher risk of AF according to TC variability quartiles was observed among individuals with COPD compared to those without COPD (*P* value for interaction = 0.0135). (Fig 4).

## 4. Discussion

The present study provides the first demonstration that high visit-to-visit TC variability was significantly associated with the risk of AF after extensive adjustments for possible confounding factors, including mean TC levels in the Korean population. In the multivariate Cox proportional hazard analysis, the risk of AF increased significantly in the highest quartile group of TC variability. There were no significant differences to AF risk between subjects in the lower three quartiles, implying that a threshold may exist at some level of TC variability.

Although the pathophysiological mechanism linking risk factors and AF has not been fully defined, inflammation has been suggested to have a major role in the development, progression, and perpetuation of AF [21]. Experimental studies have supported the pathogenic importance of inflammation regarding structural atrial damage [22]. Interestingly, several investigations have demonstrated that oscillation of blood glucose levels results in greater effects on inflammatory cytokines, oxidative stress, and endothelial dysfunction than continuous hyperglycemia [23, 24]. CVD and its risk factors as well as AF itself induce aggravation of the process of atrial structural remodeling [25]. Left atrial enlargement appears to be a significant risk factor for AF, and the proper control of blood pressure and glucose levels may be critical in the prevention of AF [26].

Hypercholesterolemia is the pivotal risk factor for CVD and mortality, but previous studies on the association between dyslipidemia and AF have had controversial results. Annoura et al. reported that low TC levels were found in patients with AF, which was described as the “cholesterol paradox” [27]. Lopez et al. also demonstrated that higher levels of TC are associated with a lower risk of AF in the Atherosclerosis Risk in Communities study [28]. However, in the Multi-Ethnic Study of Atherosclerosis and the Framingham Heart Study, low-density lipoprotein cholesterol (LDL-C) and TC were not associated with the incidence of AF [29]. Recently, in seven US and European cohort studies called AF Genetics consortium, Mendelian randomization analysis did not shown an association between AF risk and blood lipid levels including TC [30]. Furthermore, longitudinal studies regarding the association between TC variability and the development of AF are limited.

The mechanism by which TC variability affects AF can be assumed based on the previous study data concerning the effect of membrane cholesterol contents on surface expression of ultra-rapid activating delayed rectifier K current (IKur) [31]. In native atrial myocytes, cholesterol depletion increased membrane contents of IKur [31], which is a major repolarizing current in human atrium and involved in the pathophysiology of atrial fibrillation [32, 33]. Moreover, cholesterol depletion led to impairment in cardiomyocyte contractility by deregulating calcium handling, adrenergic signaling and the myofibrillar architecture [34]. Thus, severe fluctuation in blood cholesterol levels can contribute to the development of AF through changes in the cardiac membrane lipid contents. However, further experimental approaches are needed to elucidate this relationship.

Epidemiological studies reported a marked increase in AF in accordance with aging [35]. Interestingly, the subgroup analysis of our study showed a slightly higher risk of AF in individuals aged <55 years compared to those aged ≥55 years, although this interaction did not reach statistical significance (*P* = 0.0508). The elderly population tends to have more comorbidities, and chronic exposure to external stress, such as cardiometabolic risk factors, may predispose to the development of AF. However, in the present study, the association between TC variability and the risk of AF was persistent even after adjustment for age, sex, and other cardiometabolic risk parameters, such as hypertension and diabetes. Furthermore, most subgroup analyses comparing the highest and lowest quartiles of TC variability produced similar results without significant interaction, independent of risk factors apart from COPD. Although the exact underlying mechanism is not clear, individuals with COPD had a higher risk of AF according to TC variability quartiles compared to those without COPD (*P* value for interaction = 0.0135).

This study is the first to demonstrate that high TC variability was associated with an increased risk of AF. Whole genome analysis have identified numerous TC-associated genetic variants, which were associated with lipid medication use and cardiovascular risk [36, 37]. Unidentified genetic factors may affect TC variability but are not modifiable. For subjects on lipid-lowering agents, improving drug adherence could play an important role to lower the TC variability as previous study demonstrated that LDL-cholesterol variability was associated with compliance of statin [16]. Moreover, as individual lifestyle factors such as diet and exercise have a significant effect on serum cholesterol levels, maintaining a regular lifestyle may attenuate TC variability. If the association between TC variability and AF is confirmed by another studies, especially in other ethnic groups, efforts to find treatment strategies to reduce TC variability should be supported.

Our study has several limitations. First, we could not analyze various lipid profiles, including HDL-C and LDL-C, due to the lack of information. Second, patients with AF were identified according to ICD-10 codes and not confirmed by electrocardiography. Thus, the frequency of AF may have been underestimated if AF events did not result in claims. Third, even though we tried to adjust for multiple covariates that influence AF using multivariate analyses, residual or unmeasured confounding factors remained. Finally, because of the intrinsic limitations of the observational study design, we could not define a causal relationship between TC variability and AF. Recent studies have shown that variabilities in several metabolic parameters (BMI, systolic blood pressure, glucose and total cholesterol) are independent predictors for adverse outcomes, such as cardiovascular events, dementia, and mortality [14, 38, 39]. Although the association between metabolic parameter variability and health outcome is becoming robust, the underlying pathophysiology is unclear and requires additional research. Nevertheless, our study has several strengths, including a large credible database, standardized and validated by the Korean government, with detailed information concerning medications and medical diagnosis. Furthermore, this study is sufficient for the evaluation of the risk of AF in the general population setting because the median follow-up period is 8.4 years. In addition, the relationship between TC variability and AF was similar and coherent when we used three different indicators of TC variability (CV, SD, and VIM).

In conclusion, we uncovered that high TC variability is associated with an increased risk of AF by multivariate Cox proportional hazard analysis using a nationwide population-based cohort database with a sufficient follow-up period. These findings suggest that TC variability can be used as a novel predictor to stratify the risk of AF. Reducing TC variability below certain thresholds may lower the risk of AF. Further study is needed to confirm our results in other ethnic groups and to explore the mechanism of TC variability in the pathogenesis of AF.

## Supporting information

S1 Checklist Strobe checklist of items that should be included in reports of cohort studies. (DOCX)

S1 Table Baseline characteristics of the subjects according to the presence of atrial fibrillation. (DOCX)

S2 Table Baseline characteristics of subjects according to the total cholesterol variability (TC-SD). (DOCX)

S3 Table Baseline characteristics of subjects according to the total cholesterol variability (TC-VIM). (DOCX)

## Abbreviations

AF: Atrial fibrillation
ANOVA: Analysis of variance
BMI: Body mass index
CI: confidence interval
CKD: chronic kidney disease
COPD: chronic obstructive pulmonary disease
CV: coefficient of variation
CVD: Cardiovascular disease
HR: Hazard ratios
ICD-10: International Classification of Diseases, Tenth Revision
IKur: Ultra-rapid activating delayed rectifier K current
LDL-C: Low-density lipoprotein cholesterol
NHIS-HEALS: National Health Insurance Service-National Health Screening Cohort
OSA: obstructive sleep apnea
SD: standard deviation
TC: Total cholesterol
VIM: variability independent of the mean
