# Supplementary material for: Total cholesterol variability and risk of atrial fibrillation: A nationwide population-based cohort study
Source: PLoS One. 2019 Apr 24;14(4):e0215687. doi: 10.1371/journal.pone.0215687 (PMC6481829; doi:10.1371/journal.pone.0215687)
Supplement: S1 Table — (DOCX) [file pone.0215687.s002.docx]

**S1 Table.** Baseline characteristics of the subjects according to the presence of atrial fibrillation.

|  | Overall | Without AF | With AF | *P* value |
| --- | --- | --- | --- | --- |
| N | 160165 | 156281 | 3884 |  |
| Age (years) | 55.9±8.8 | 55.7±8.7 | 62.5±9.7 | <.0001 |
| Sex (male) (n, %) | 94312 (58.9) | 91723 (58.7) | 2589 (66.7) | <.0001 |
| Body mass index (kg/m2) | 24.0±2.9 | 23.9±2.8 | 24.4±3.0 | <.0001 |
| Systolic BP (mmHg) | 125.7±15.8 | 125.7±15.7 | 129.5±16.2 | <.0001 |
| Diastolic BP (mmHg) | 78.3±10.2 | 78.3±10.2 | 79.5±10.3 | <.0001 |
| Aspartate transaminase (IU/L) | 26.3±15.9 | 26.2±15.8 | 27.8±17.4 | <.0001 |
| Alanine transaminase (IU/L) | 25.3±19.4 | 25.3±19.4 | 25.2±17.9 | 0.9015 |
| γ-glutamyl transferase (IU/L) | 38.5±50.4 | 38.3±50.0 | 45.5±64.5 | <.0001 |
| Fasting plasma glucose (mmol/L) | 99.0±25.9 | 98.9±25.8 | 102.0±29.2 | <.0001 |
| Mean TC (mg/dL) | 198.8±30.5 | 198.9±30.4 | 196.4±30.7 | <.0001 |
| TC variability |  |  |  |  |
| TC-CV (%) | 10.31±5.87 | 10.29±5.85 | 11.21±6.54 | <.0001 |
| TC-SD (IU/L) | 20.53±12.65 | 20.49±12.62 | 22.05±13.65 | <.0001 |
| TC-VIM (%) | 20.45±11.88 | 20.41±11.84 | 22.12±13.08 | <.0001 |
| Current smoker (n, %) | 32538 (20.3) | 31784 (20.3) | 754 (19.4) | 0.1671 |
| Alcohol consumption (n, %) | 68784 (42.9) | 67153 (43.0) | 1631 (42.0) | 0.2358 |
| Regular exercise (n, %) | 16450 (10.3) | 15978 (10.2) | 472 (12.2) | 0.0001 |
| Income (lower 10%) (n, %) | 12800 (8.0) | 12443 (8.0) | 357 (9.2) | 0.0065 |
| Hypertension (n, %) | 96055 (60.0) | 93078 (59.6) | 2977 (76.6) | <.0001 |
| Dyslipidemia (n, %) | 55769 (34.8) | 54284 (34.7) | 1485 (38.2) | <.0001 |
| Diabetes (n, %) | 28436 (17.8) | 27523 (17.6) | 913 (23.5) | <.0001 |
| Heart failure (n, %) | 3836 (2.4) | 3505 (2.2) | 331 (8.5) | <.0001 |
| Ischemic heart disease (n, %) | 21821 (13.6) | 20752 (13.3) | 1069 (27.5) | <.0001 |
| Cerebrovascular disease (n, %) | 15479 (9.7) | 14749 (9.4) | 730 (18.8) | <.0001 |
| Chronic kidney disease (n, %) | 772 (0.5) | 724 (0.5) | 48 (1.2) | <.0001 |
| Thyroid disorder (n, %) | 12537 (7.8) | 12147 (7.8) | 390 (10.0) | <.0001 |
| Chronic obstructive pulmonary disease (n, %) | 10094 (6.3) | 9556 (6.1) | 538 (13.9) | <.0001 |
| Obstructive sleep apnea (n, %) | 525 (0.3) | 508 (0.3) | 17 (0.4) | 0.2382 |

*P* value derived using ANOVA and χ^2^ tests.

Data are expressed as mean ± SD, or n (%).

Abbreviations: AF, atrial fibrillation; BP, blood pressure; CV, coefficients of variance; SD, standard deviation; TC, total cholesterol; VIM, variability independent of the mean.
