# Supplementary material for: Total cholesterol variability and risk of atrial fibrillation: A nationwide population-based cohort study
Source: PLoS One. 2019 Apr 24;14(4):e0215687. doi: 10.1371/journal.pone.0215687 (PMC6481829; doi:10.1371/journal.pone.0215687)
Supplement: S2 Table — (DOCX) [file pone.0215687.s003.docx]

**S2 Table.** Baseline characteristics of subjects according to the total cholesterol variability (TC-SD)

|  | Q1 | Q2 | Q3 | Q4 | *P*-value |
| --- | --- | --- | --- | --- | --- |
| N | 40041 | 40041 | 40035 | 40048 |  |
| Age (years) | 55.8±8.9 | 54.9±8.4 | 55.4±8.5 | 57.5±9.0 | <.0001 |
| Sex (male) (n, %) | 23711 (59.2) | 25244 (63.0) | 24006 (60.0) | 21351 (53.3) | <.0001 |
| Body mass index (kg/m2) | 23.8±2.9 | 23.8±2.8 | 24.0±2.8 | 24.2±2.9 | <.0001 |
| Systolic BP (mmHg) | 124.8±15.6 | 125.2±15.5 | 125.9±15.7 | 127.1±16.1 | <.0001 |
| Diastolic BP (mmHg) | 77.8±10.2 | 78.3±10.1 | 78.5±10.2 | 78.8±10.3 | <.0001 |
| Aspartate transaminase (IU/L) | 25.5±12.6 | 25.8±14.8 | 26.4±16.1 | 27.4±19.1 | <.0001 |
| Alanine transaminase (IU/L) | 24.1±16.8 | 24.9±19.7 | 25.5±18.7 | 26.7±21.8 | <.0001 |
| γ-glutamyl transferase (IU/L) | 34.3±41.1 | 37.1±45.1 | 39.5±50.2 | 43.1±62.3 | <.0001 |
| Fasting plasma glucose (mmol/L) | 97.3±23.0 | 97.8±23.9 | 98.8±25.5 | 101.9±30.2 | <.0001 |
| Mean TC (mg/dL) | 190.2±29.0 | 194.5±28.5 | 200.0±28.7 | 210.6±31.5 | <.0001 |
| TC variability |  |  |  |  |  |
| TC-CV (%) | 4.51±1.64 | 7.93±1.44 | 10.98±1.91 | 17.82±5.78 | <.0001 |
| TC-SD (IU/L) | 8.39±2.76 | 15.11±1.65 | 21.53±2.22 | 37.09±13.13 | <.0001 |
| TC-VIM (%) | 8.69±2.95 | 15.44±2.09 | 21.64±2.78 | 36.02±11.66 | <.0001 |
| Current smoker (n, %) | 7838 (19.6) | 8756 (21.9) | 8515 (21.3) | 7429 (18.6) | <.0001 |
| Alcohol consumption (n, %) | 17312 (43.2) | 18328 (45.8) | 17618 (44.0) | 15526 (38.8) | <.0001 |
| Regular exercise (n, %) | 4234 (10.6) | 3859 (9.6) | 3948 (9.9) | 4409 (11.0) | <.0001 |
| Income (lower 10%) (n, %) | 2868 (7.2) | 2932 (7.3) | 3293 (8.2) | 3707 (9.3) | <.0001 |
| Hypertension (n, %) | 22038 (55.0) | 23132 (57.8) | 24221 (60.5) | 26664 (66.6) | <.0001 |
| Dyslipidemia (n, %) | 5766 (14.4) | 8804 (22.0) | 14218 (35.5) | 26981 (67.4) | <.0001 |
| Diabetes (n, %) | 5654 (14.1) | 6292 (15.7) | 7025 (17.5) | 9465 (23.6) | <.0001 |
| Heart failure (n, %) | 820 (2.0) | 733 (1.8) | 878 (2.2) | 1405 (3.5) | <.0001 |
| Ischemic heart disease (n, %) | 4779 (11.9) | 4616 (11.5) | 4954 (12.4) | 7472 (18.7) | <.0001 |
| Cerebrovascular disease (n, %) | 3402 (8.5) | 3198 (8.0) | 3606 (9.0) | 5273 (13.2) | <.0001 |
| Chronic kidney disease (n, %) | 152 (0.4) | 152 (0.4) | 163 (0.4) | 305 (0.8) | <.0001 |
| Thyroid disorder (n, %) | 2772 (6.9) | 2662 (6.6) | 3051 (7.6) | 4052 (10.1) | <.0001 |
| Chronic obstructive pulmonary disease (n, %) | 2467 (6.2) | 2139 (5.3) | 2390 (6.0) | 3098 (7.7) | <.0001 |
| Obstructive sleep apnea (n, %) | 95 (0.2) | 149 (0.4) | 134 (0.3) | 147 (0.4) | 0.0024 |

*P* value derived using ANOVA and χ^2^ tests.

Data are expressed as mean ± SD, or n (%).

BP = blood pressure; CV = coefficients of variance; SD = standard deviation; TC = total cholesterol; VIM = variability independent of the mean
