# Supplementary material for: Total cholesterol variability and risk of atrial fibrillation: A nationwide population-based cohort study
Source: PLoS One. 2019 Apr 24;14(4):e0215687. doi: 10.1371/journal.pone.0215687 (PMC6481829; doi:10.1371/journal.pone.0215687)
Supplement: S3 Table — (DOCX) [file pone.0215687.s004.docx]

**S3 Table.** Baseline characteristics of subjects according to the total cholesterol variability (TC-VIM)

|  | Q1 | Q2 | Q3 | Q4 | *P*-value |
| --- | --- | --- | --- | --- | --- |
| N | 40041 | 40041 | 40042 | 40041 |  |
| Age (years) | 55.8±8.9 | 54.8±8.3 | 55.4±8.5 | 57.5±9.1 | <.0001 |
| Sex (male) (n, %) | 23423 (58.5) | 25238 (63.0) | 23993 (59.9) | 21658 (54.1) | <.0001 |
| Body mass index (kg/m2) | 23.9±2.9 | 23.9±2.8 | 23.9±2.8 | 24.1±2.9 | <.0001 |
| Systolic BP (mmHg) | 125.1±15.6 | 125.3±15.5 | 125.8±15.7 | 126.8±16.1 | <.0001 |
| Diastolic BP (mmHg) | 77.9±10.2 | 78.4±10.2 | 78.5±10.2 | 78.6±10.3 | <.0001 |
| Aspartate transaminase (IU/L) | 25.4±12.5 | 25.8±14.0 | 26.3±16.1 | 27.6±19.8 | <.0001 |
| Alanine transaminase (IU/L) | 24.2±16.6 | 24.9±19.4 | 25.4±18.8 | 26.6±22.2 | <.0001 |
| γ-glutamyl transferase (IU/L) | 34.8±41.5 | 37.2±43.8 | 39.1±49.8 | 43.0±63.2 | <.0001 |
| Fasting plasma glucose (mmol/L) | 97.6±23.3 | 97.8±23.8 | 98.7±25.6 | 101.7±30.0 | <.0001 |
| Mean TC (mg/dL) | 195.3±29.7 | 196.7±29.4 | 198.7±29.9 | 204.5±32.0 | <.0001 |
| TC variability |  |  |  |  |  |
| TC-CV (%) | 4.39±1.47 | 7.80±0.97 | 10.95±1.29 | 18.10±5.55 | <.0001 |
| TC-SD (IU/L) | 8.48±2.89 | 15.21±2.10 | 21.56±2.87 | 36.86±13.33 | <.0001 |
| TC-VIM (%) | 8.59±2.82 | 15.34±1.63 | 21.62±2.15 | 36.24±11.47 | <.0001 |
| Current smoker (n, %) | 7803 (19.5) | 8794 (22.0) | 8410 (21.0) | 7531 (18.8) | <.0001 |
| Alcohol consumption (n, %) | 17234 (43.0) | 18336 (45.8) | 17608 (44.0) | 15606 (39.0) | <.0001 |
| Regular exercise (n, %) | 4265 (10.7) | 3917 (9.8) | 3893 (9.7) | 4375 (10.9) | <.0001 |
| Income (lower 10%) (n, %) | 2891 (7.2) | 2959 (7.4) | 3293 (8.2) | 3657 (9.1) | <.0001 |
| Hypertension (n, %) | 22312 (55.7) | 23248 (58.1) | 24058 (60.1) | 26437 (66.0) | <.0001 |
| Dyslipidemia (n, %) | 7340 (18.3) | 10136 (25.3) | 14086 (35.2) | 24207 (60.5) | <.0001 |
| Diabetes (n, %) | 5717 (14.3) | 6256 (15.6) | 7021 (17.5) | 9442 (23.6) | <.0001 |
| Heart failure (n, %) | 803 (2.0) | 757 (1.9) | 860 (2.1) | 1416 (3.5) | <.0001 |
| Ischemic heart disease (n, %) | 4801 (12.0) | 4547 (11.4) | 4993 (12.5) | 7480 (18.7) | <.0001 |
| Cerebrovascular disease (n, %) | 3410 (8.5) | 3199 (8.0) | 3599 (9.0) | 5271 (13.2) | <.0001 |
| Chronic kidney disease (n, %) | 154 (0.4) | 148 (0.4) | 164 (0.4) | 306 (0.8) | <.0001 |
| Thyroid disorder (n, %) | 2770 (6.9) | 2633 (6.6) | 3050 (7.6) | 4084 (10.2) | <.0001 |
| Chronic obstructive pulmonary disease (n, %) | 2445 (6.1) | 2115 (5.3) | 2399 (6.0) | 3135 (7.8) | <.0001 |
| Obstructive sleep apnea (n, %) | 104 (0.3) | 138 (0.3) | 130 (0.3) | 153 (0.4) | 0.0218 |

*P* value derived using ANOVA and χ^2^ tests.

Data are expressed as mean ± SD, or n (%).

BP = blood pressure; CV = coefficients of variance; SD = standard deviation; TC = total cholesterol; VIM = variability independent of the mean
